# Single dynamic structured light navigation for minimally invasive cervical pedicle screw insertion: Achieving complex design and precise puncture

**DOI:** 10.1371/journal.pone.0346713

**Published:** 2026-05-11

**Authors:** Xuanhuang Chen, Xiaoxia Huang, Guodong Zhang, Kaijun Li, Qi Liu, Zaopeng He, Weihong Xu

**Affiliations:** 1 Department of Spinal Surgery, the First Affiliated Hospital, Fujian Medical University, Fuzhou, China; 2 Department of Orthopedics, the Affiliated Hospital of Putian University, Putian, China; 3 Department of Orthopedic Surgery, National Regional Medical Center, Binhai Campus of the First Affiliated Hospital, Fujian Medical University, Fuzhou, China; 4 Fujian Orthopedic Bone and Joint Disease and Sports Rehabilitation Clinical Medical Research Center, the First Affiliated Hospital, Fujian Medical University, Fuzhou, China; 5 Lecong Hospital of Shunde, Foshan, China; 6 The Graduate School of Fujian Medical University, Fuzhou, China; Columbia University Vagelos College of Physicians and Surgeons, UNITED STATES OF AMERICA

## Abstract

**Aim:**

The aim of this study was to explore the preoperative design and intraoperative navigation for minimally invasive cervical pedicle punctures and to validate the feasibility and accuracy of a single dynamic structured light navigation system.

**Methods:**

Nine bovine cervical spine bone models were created for preoperative CT scanning and three-dimensional reconstruction. Preoperative segmentation of the vertebral bodies and design of the navigation channels were conducted. During the procedure, structured light scanning was employed to capture the intraoperative scene and facilitate registration. The navigation channel was adjusted to align with the preoperative design. A 2.0 mm stainless steel Kirschner wire was subsequently inserted into each pedicle through separate punctures. The position of the Kirschner wire was evaluated via the Neo classification system. Intraoperative root mean square (RMS) values and registration accuracy were recorded. The deviations and angles of the preoperative and postoperative entry and exit points were calculated on the basis of the three-dimensional coordinates of these points. Statistical analyses were performed to assess differences in the X, Y, and Z coordinate values of the entry and exit points before and after the procedure.

**Results:**

A total of 124 Kirschner wires were inserted, and the Neo classification evaluation for all models was Grade 0, with a puncture accuracy of 100%. There was high consistency between the postoperative Kirschner wire trajectories and the preoperative design. The average intraoperative RMS and registration accuracies were 0.424 ± 0.12 mm and 0.01 ± 0.008 mm, respectively. The preoperative and postoperative entry point deviation was 0.904 ± 0.419 mm, and the exit point deviation was 1.253 ± 0.607 mm. The entry and exit point deviation angle was 0.918 ± 0.421°. The deviation may be related to bone surface factors.

**Conclusion:**

A single dynamic structured light navigation system is capable of performing simultaneous preoperative complex design, intraoperative scene scanning, static registration, and dynamic tracking of piercing instruments, thereby enabling high-precision minimally invasive punctures.

## Introduction

Cervical spondylosis, deformities, tumors, and trauma are considered to significantly impact the quality of life and may lead to disability in severe cases. Patients may require treatment through posterior cervical pedicle screw internal fixation. Owing to the complex anatomy, proximity to the vertebral artery, nerves, and spinal cord, and thick muscle and fat tissue at the back of the neck, the low fault tolerance associated with small posterior incision screw placement results in high surgical risks and significant challenges. Minimally invasive spine surgery (MIS) has become widely adopted due to its minimal tissue disruption. Intelligent planning can automatically identify the target vertebral body and dynamically plan the trajectory, specifications, and insertion depth of the pedicle screw based on the unique bone structure, shape, and injury characteristics of each vertebra. The development of MIS fixation technology has been gradual, with side block fixation being predominantly utilized in the early stages [1].

Numerous scholarly investigations [2–4]have documented perforation rates spanning from a modest 3.8% to a significant 27% in the context of manually inserted and fluoroscopically guided cervical pedicle screws (CPS). CPS perforation can lead to neurological or vascular damage, with neurological complications occurring in up to 3.7% of cases. The incidence of vertebral artery injury ranges from 4% to 8% [5], particularly when the lateral distance from the pedicle to the vertebral artery is as narrow as 0.89 mm [6]. Despite ongoing advancements in counterpinning techniques and improvements in the precision of CPS placement in the upper cervical spine (C1–C2), the accuracy of CPS placement in the lower cervical spine (C3–C7) remains highly variable, with reported success rates ranging from as low as 16.8% to as high as 97% [7]. With the advent of computer navigation technology, the use of navigational CPS fixation facilitated the insertion of larger-diameter and longer screws [8,9], thereby ensuring biomechanically stable fixation. This technique has proven especially effective in restoring cervical lordosis [10] while significantly minimizing complications such as surgical site fractures, infections, and the need for cervical revision surgeries [11]. Both computer-aided navigation and robotic guidance outperform traditional freehand techniques in terms of the accuracy of CPS placement. Moreover, advanced systems, from O-arm imaging to complex robotic navigation, further increase the precision of screw placement [12–14].

Near-infrared light navigation has become the mainstream navigation method because of its well-established clinical applications [15,16]. Owing to the significant deviation angle required for cervical spine placement, the line of sight between the navigation marker and the camera is often obstructed, leading to potential errors [17]. The cervical vertebrae exhibit a high degree of intervertebral motion, which may lead to a mismatch between the actual anatomical structure and the displayed model during surgery [18]. This motion can also cause relative displacement between the surgical vertebra and the reference frame or result in loosening of the reference frame due to screw placement, all of which can contribute to navigation errors [19]. Near-infrared light navigation requires the placement of marker balls on rigid objects, such as imaging equipment, surgical instruments, patients, and reference frames, which can be tracked. The limited number of matching points for these markers may be a key factor preventing further improvement in their mechanical accuracy [20].

Sugimoto *et al*. [21] reported an average degree of cervical rotation of 10.6° during CPS insertion. Even with intraoperative 3D-based navigation, this vertebral rotation can be a significant cause of CPS dislocation. Intraoperatively, multiple imaging scans and adjustments may be needed, which could result in increased additional radiation exposure to the patient. The reference frame should be positioned in a stable location that not only avoids interference with the surgical area but also prevents inadvertent contact, which could lead to inaccurate navigation [22,23]. The new marker-free 3D structured light registration method is simple, improves clinical efficiency, provides sufficient accuracy and safety, and fully meets the clinical requirements for navigational puncture [24]. A 7D surgical system based on structured light navigation has been clinically used for CPS nail placement. However, the device relies solely on static structured light for navigation registration [25,26], whereas the nail placement instrument still requires near-infrared light positioning and tracking. This reduces the convenience for the operator, affects the actual accuracy of nail placement, and increases the theoretical complexity of navigation.

The study innovatively applies dynamic structured light to percutaneous minimally invasive cervical pedicle puncture navigation, distinguishing it from traditional near-infrared light navigation and mixed navigation methods that combine static structured light registration with near-infrared light tracking for nail placement instruments. The navigation principle uses a single structured-light camera throughout the procedure, continuously scanning the surgical scene. It collects surface point clouds from the actual surgical space and registers them with surface point clouds from medical images (derived from CT data). Using the resulting transformation, the entire medical image, including bone and other anatomy, can be mapped into the surgical space to localize the target. The structured light also captures surface points from the surgical instruments in real time, enabling dynamic identification and tracking of pedicle-screw instruments and guiding puncture. Thus, the surgery proceeds within a single, dynamic structured-light navigation system. This study accomplished software development, instrument design, and customization, successfully achieving preoperative planning and precise intraoperative puncture in cervical spine surgery, thereby providing both theoretical insights and practical references for the development of new computer navigation methods.

## Materials and methods

### Software and hardware

**Fig 1:**
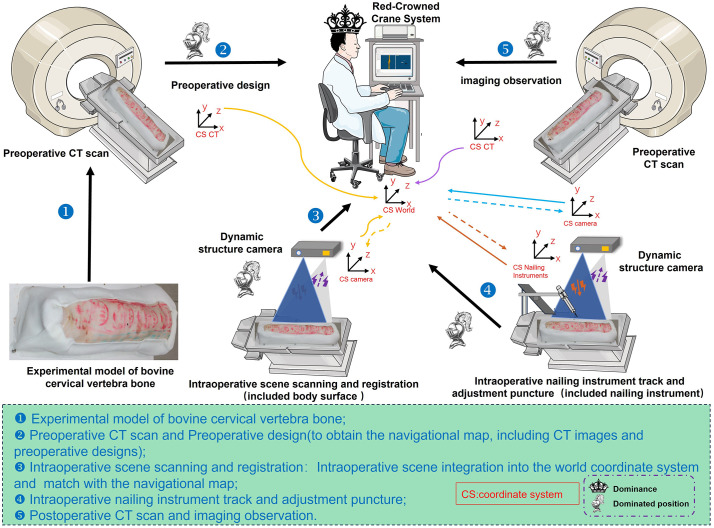
Schematic diagram of the experimental procedure.

RCCS V1.0 navigation software (Red-Crowned Crane System, self-developed); 3D structured light camera supporting application software (MPSizectorS SDK V2.21, MEGA PHASE, China); 3D Slicer-5.6.2（National Institutes of Health，America）; 3D structured light camera (Sizector S028800, MEGA PHASE, China); spiral CT (INGENUITY CORE 128 CT, Philips, Netherlands). A schematic diagram of the methodological workflow is shown in Fig 1.

### Instruments and consumables

**Fig 2:**
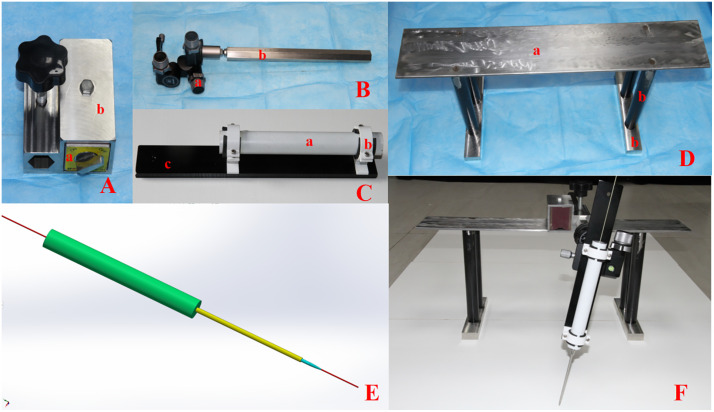
Precision machining instruments. **A** Magnetic base: a magnetic seat, b shell; **B** Universal head and longitudinal rod: a universal joint, b adjustment vertical rod; **C** Navigation drill sleeve and adapter plate:a navigation drill sleeve, b fixed clip, c adapter plate; **D** Gantry crane structure:a transverse adjustable magnetic base plate, b supports; **E** Puncture instrument design drawing; **F** Adjust the puncture instrument.

Our team has designed and precisely manufactured custom navigation drill sleeves, adjustment instruments, and puncture devices. The navigation drill sleeve is made of an aluminum alloy hollow cylindrical tube (25 mm * 6.05 mm * 200 mm). The adjustment instruments include a gantry structure (with dual side supports, a transverse adjustable magnetic base plate), a magnetic seat, an adjustment vertical rod, an adapter plate, and a universal joint. The puncture device consists of an outer sleeve (6 mm * 4.05 mm * 300 mm) and an inner sleeve (4 mm * 2.05 mm * 300 mm), both of which are made of titanium alloy and are precisely fitted together as coaxial sleeves to form a puncture tube (Fig 2).

Bovine cervical vertebra, Pig skin (with a fat layer approximately 20 mm thick), Polymer polyurethane bandage, φ2.0 mm stainless steel Kirschner needle, φ1.5 mm titanium Kirschner needle, Wooden bottom plate; Wood chips and cloth bags.

### Experimental model of bovine cervical vertebra bone

**Fig 3:**
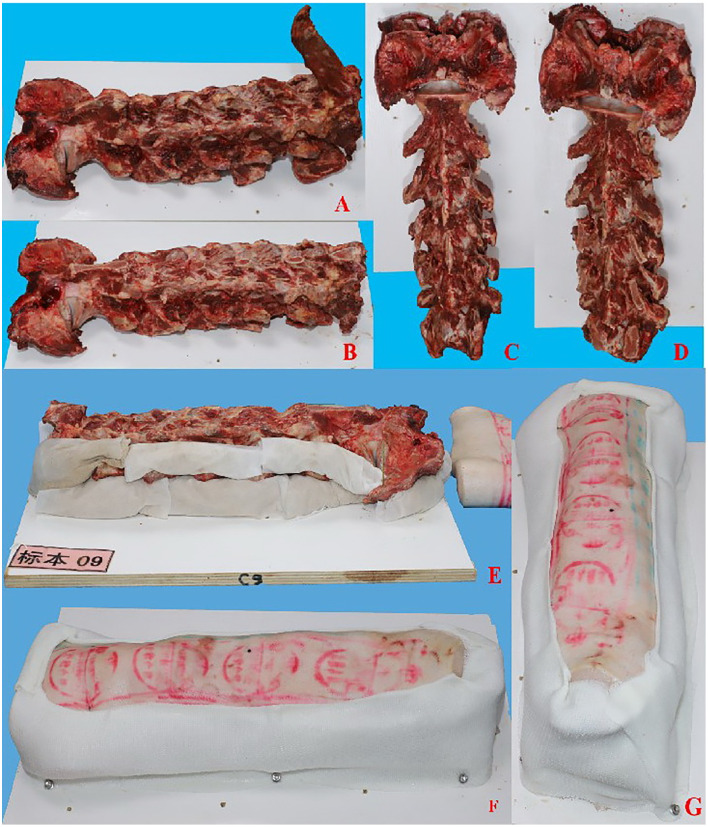
Experimental model of bovine cervical vertebra. **A** Lateral view of bovine cervical vertebra; **B** Lateral view of bovine cervical vertebra after treatment; **C** Ventral view of bovine cervical vertebra; **D** Ventral view of bovine cervical vertebrae after treatment; **E** Bovine bone and pig skin after fixed molding; **F** Lateral view of bovine bone after wrapping with polymer bandages; **G** Dorsal view of beef bone after wrapping with polymer bandage.

The bovine cervical vertebrae were covered with pig skin. The spinous processes and cervical rib processes of the bovine cervical vertebrae were truncated, with the spinous processes facing upward. Several titanium Kirschner wires were used to securely fix the bovine bone onto a wooden base. The transverse processes and the area around the vertebral body were densely packed with cloth bags filled with sawdust. Pigskin was then applied over the bovine bone and the cloth bags, and titanium Kirschner wires were used for fixation (Fig 3A-E). The polymer polyurethane bandage was wrapped, the surgical field was exposed above, and the bandage was fixed to the wooden base plate with screws below (Fig 3F-G).

### Preoperative CT scan and Preoperative design

The CT scanning parameters were as follows: tube voltage of 120 kV, tube current of 30 mA, pitch of 0.625 mm, and slice thickness of 0.67 mm. After interpolation, DICOM-formatted images with a resolution of 0.325 mm were output. These images were imported into 3D Slicer, where thresholding, region growing, contour line calculation, and filling were applied. A 3D reconstruction of the experimental model’s surface and skeletal structures was generated, and the output was saved as an STL file.

**Fig 4:**
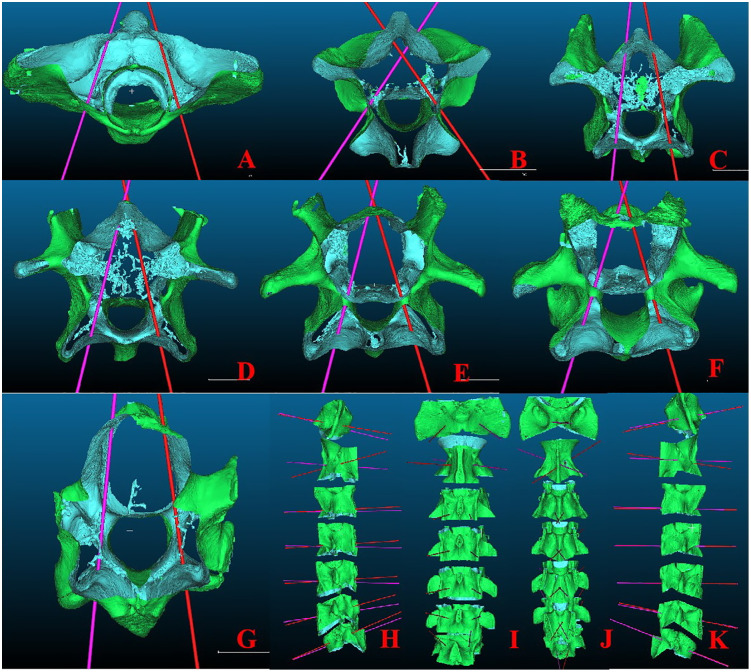
Preoperative vertebral body segmentation and channel design. **A-G** Views of C1-7 vertebral bodies and channels from below; **H** Views of vertebral bodies and channels from the left side; **I** Views of vertebral bodies and channels from the posterior side； **J** Views of vertebral bodies and channels from the anterior side; **K** Views of vertebral bodies and channels from the right side.

RCCS features a dual-window interface. The STL file was imported into the left window of the RCCS for further operation. Vertebral body segmentation and channel design: Click on the “segment” and, on the lateral surface of the vertebral body, trace along the superior endplate, the superior margins of the bilateral articular processes, and the inferior endplate. Then, “Segment In” and “Confirm Segmentation” are clicked to independently segment the vertebral body. The vertebral body is selected, and then the “Primitive Factory and Cylinder” is clicked on. The radius is set to 1 mm, and the height is 300 mm. Click on “Translate/Rotate” to move the cylinder to the left pedicle long axis. The cylinder was rotated from all four sides to ensure that it was positioned correctly without penetrating any holes. Then, “Clone the selected entities” is clicked to duplicate the cylinder with the same specifications. The “Translate/Rotate” command was used to move the cloned cylinder to the appropriate position along the long axis of the right pedicle. Using the same method, the remaining vertebral bodies were sequentially segmented, and the respective preoperative channels were designed. The surface, bones, individual vertebral bodies, and preoperative channel designs are combined into a single folder, named the “ navigational map” (Fig 4).

### Intraoperative Scene Scanning and registration

**Fig 5:**
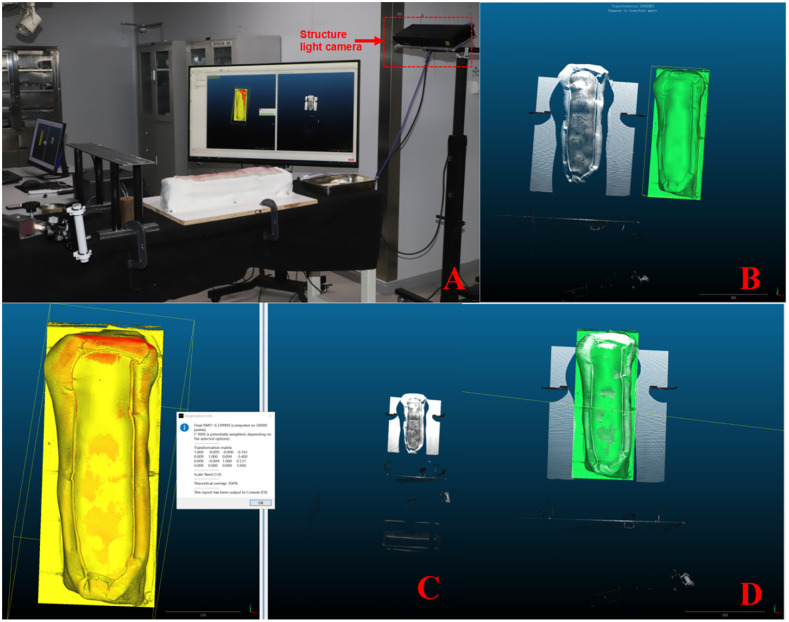
Positioning and Registration. **A** Positioning of the experimental model and structured light camera; **B** Point cloud extraction; **C** Fine registration completed to obtain the root mean square (RMS) value; **D** The “ navigational map “is moved to the intraoperative scene point cloud position.

Experimental model positioning. The experimental model was placed on the operating bed, and the wooden bottom plate was fixed to the operating bed (Fig 5A). The structured light camera parameters are set to precision mode, pixel merging is enabled, an automatic continuous trigger is used, external exposure is disabled, white light-enabled, and RGB-enabled, a fixed-point 3D point cloud is applied for postprocessing, and a mixed texture mode with a polygon fill is chosen for rendering. The structured light camera was positioned above and in front of the experimental model. “RunSdk” is clicked on the right window of the RCCS to activate the structured light camera, ensuring that both the experimental model and the navigation drill sleeve are within the scanning range of the structured light camera (Fig 5A). For rough register and point cloud editing, sequentially click “Start,” “Stop,” and “Capture” in the right window of RCCS to capture a frame of the current intraoperative scene’s point cloud and transfer it to the left window (Fig 5B). Select the point cloud and the surface model, then click “Aligns two clouds by picking (at least 4) equivalent point pairs” to move the point cloud into initial alignment with the surface model. Next, “Segment” is clicked, and the “SOR” filter is used to crop the point cloud and remove noise points.

For the Finely Register, select the point cloud and the surface model, then click “Finely registers already (roughly) aligned entities (clouds or meshes).” The “cloud registration” window pops up. The reference is set to the surface model, and the aligned entity is set to the point cloud. According to the Iterated Closest Points (ICP) algorithm, the point cloud is further aligned with the surface model, at which point the RMS value for the finely registered process can be obtained (Fig 5C). For registration accuracy measurement, the point cloud and the surface model are selected, and then the “Compute cloud/mesh distance” is clicked to measure the distance between the two entities. The resulting distance value represents the registration accuracy. Inverse matrix motion “ navigational map “. The navigation software automatically calculates the matrix based on the relevant Equation.The matrix after point cloud registration in the property bar is copied, the “ navigational map “ is selected, Edit/Apply transformation (Ctrl T) is clicked, the matrix parameters in the application transformation matrix box are pasted, and the inverse transformation transformation transformation is checked.The”navigational map” was moved to the spot cloud position in the middle of the operation and entered the world coordinate system of 3D structured light (Fig 5D).

### Intraoperative nailing instrument track and adjustment puncture

**Fig 6:**
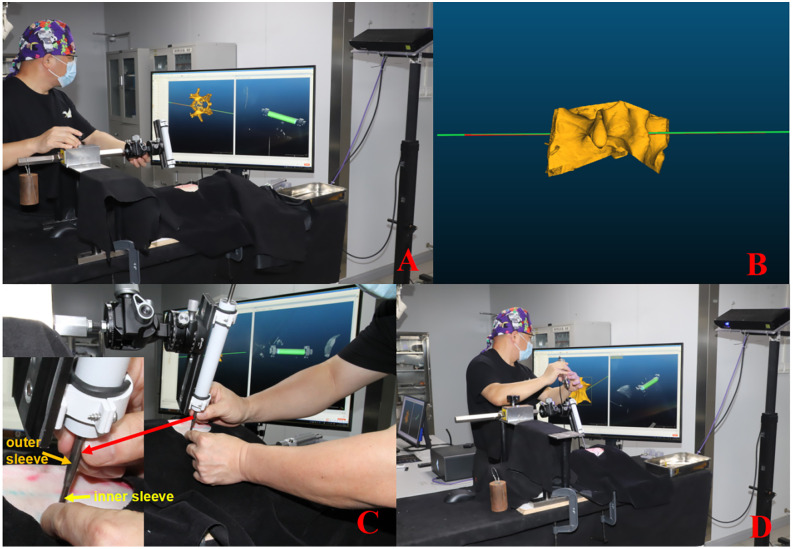
Intraoperative Needle Adjustment. **A** Intraoperative adjustment from the top view; **B** Intraoperative adjustment from the side view; **C** Insertion of the needle guide; **D** Needle puncture along the guide.

The navigation channel is adjusted. Install and secure the instrument on the operating table, then click “RunSdk” to switch the structured light camera’s working mode to either Standard or Fast. “Settings” was selected to adjust the navigation channel diameter to 2 mm, the length to 800 mm, and the instrument diameter to 25 mm. Directly scan the navigation drill sleeve via structured light. Click “Start” followed by “Navigation.” The point cloud and the fitted cylinder are displayed in the right window in real time, whereas the corresponding navigation channel, derived from the fitted cylinder, is simultaneously shown in the left window. The direction is adjusted via the universal gimbal, and the position is fine-tuned with the vertical rod to align the navigation channel with the designed nail path. The entry and exit points of the needle were observed via rotation (Fig 6A and B).

The puncture sleeve was inserted into the navigation drill sleeve, and a small skin incision was made for entry. The φ2.0 mm Kirschner needle was then advanced through the inner sleeve into the vertebral pedicle. After penetrating the anterior edge of the vertebral body, the Kirschner needle was shortened, completing the pedicle puncture on one side. The procedure was repeated for the other pedicles until all were successfully punctured (Fig 6C and D).

### Postoperative CT scan

**Fig 7:**
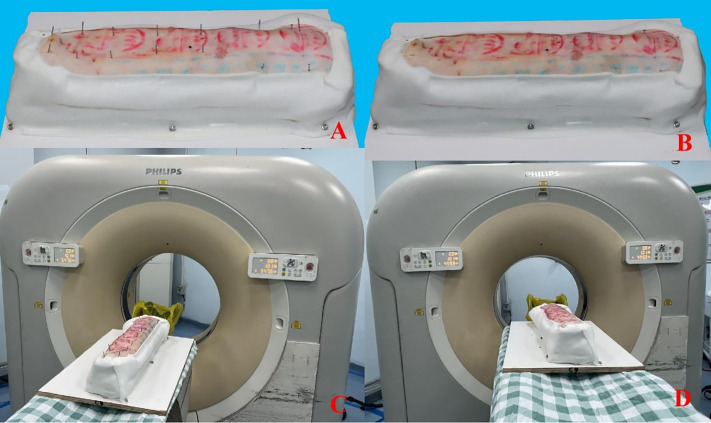
Postoperative CT Scan. **A** Experimental model with the needle in place; **B** Experimental model without the needle; **C** CT scan of the experimental model with the needle in place; **D** CT scan of the experimental model without the needle.

Postoperatively, a CT scan was performed on the experimental model both with Kirschner wires in place and after their removal. The scanning parameters and the method for 3D reconstruction were consistent with those described previously (Fig 7).

### Imaging observation, Neo classification evaluation

The preoperative bone was registered with both the postoperative bone with the Kirschner needle in place and the postoperative bone after the needle was removed. CT images of the bone and Kirschner needle were analyzed [27], and Neo classification was applied to assess the positioning of the Kirschner needle. The preoperative design channel and position of the Kirschner needle, as well as the distribution of the Kirschner needle on the bone postoperation, were observed.

### Experimental Data Collection

**Fig 8:**
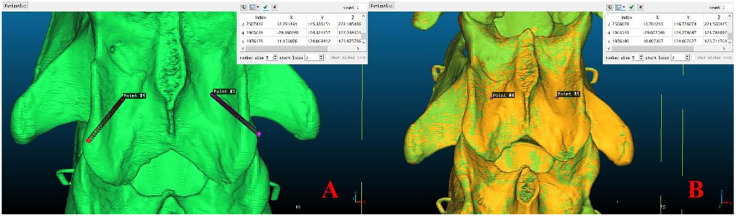
Preoperative Needle Entry and Exit Points on the Skeleton. **A** Marking of the needle entry points for the preoperative designed channels; **B** Marking of the Kirschner wire exit points.

The RMS and registration accuracy data were collected during the procedure, and the mean values were calculated and are presented as the means ± standard deviations. The entry and exit points of the needle were identified on the preoperative bone. The center points of the intersection between each designed channel and the entry/exit needle surface on the preoperative bone were considered the entry and exit points for the preoperatively designed channel. The center points of the entry/exit holes on the postoperative bone were taken as the entry and exit points of the Kirschner needle. The 3D coordinate values of each point were then output (Fig 8).

The 3D coordinate values of the entry and exit points before and after the procedure were collected to calculate the displacement of the entry and exit points and the offset angle between the preoperatively designed channel and the Kirschner needle. The differences in the coordinates (X, Y, Z) of the entry and exit points before and after the procedure were compared.

### Statistical analysis

Statistical analysis was performed via IBM SPSS Statistics 24.0. Quantitative data that followed a normal distribution are expressed as the mean ± standard deviation, and a t test was used. For data that did not follow a normal distribution, the median (M) and interquartile range (Q), expressed as M (Q1, Q3), were used, and the rank sum test was applied. In all the statistical analyses, statistical significance was established at ***p*** < 0.05.

## Results

### Neo classification evaluation

**Fig 9:**
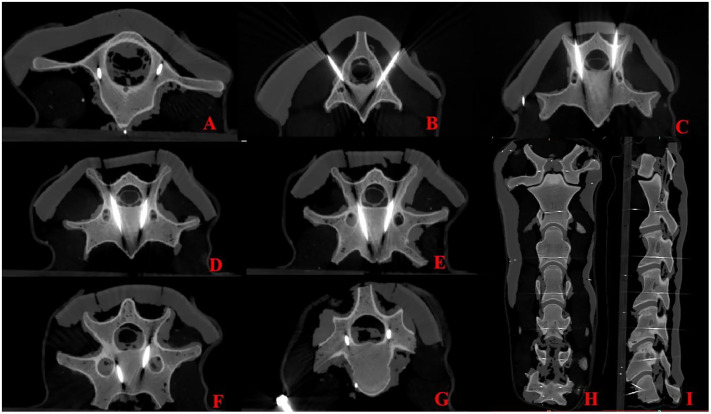
Postoperative CT Images of the Needle-Included Skeleton and Kirschner Wire. **A-G** Postoperative vertebrae (C1-C7) and Kirschner wire in the horizontal plane; **H** Vertebrae and Kirschner wire in the coronal plane.; **I** Vertebrae and Kirschner wire in the sagittal plane.

**Fig 10:**
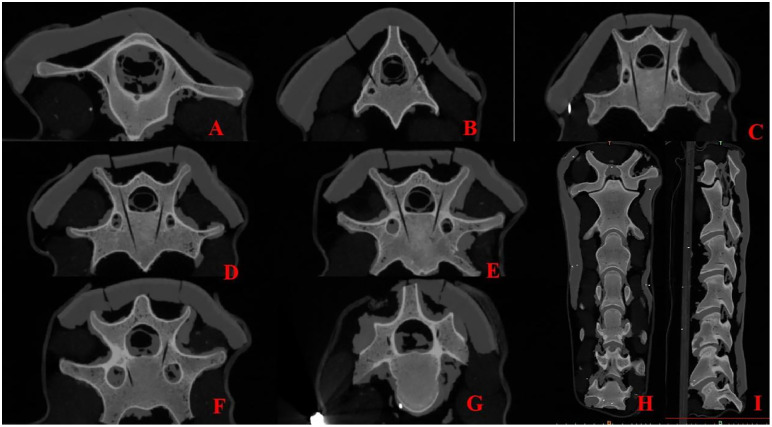
Postoperative CT Images of the Needle-Removed Skeleton. **A-G** Postoperative vertebrae (C1-C7) in the horizontal plane； **H** Vertebrae in the coronal plane； **I** Vertebrae in the sagittal plane.

A total of 124 Kirschner wires were inserted into the 9 experimental models. Postoperative CT images of the bones and Kirschner wires were obtained. According to the Neo classification, all Kirschner wires were classified as grade 0, indicating a 100% accuracy rate for pedicle screw insertion via the cervical vertebrae (Figs 9 and 10).

### The postoperative CT images obtained with the Kirschner needle were in good agreement with the preoperative design

**Fig 11:**
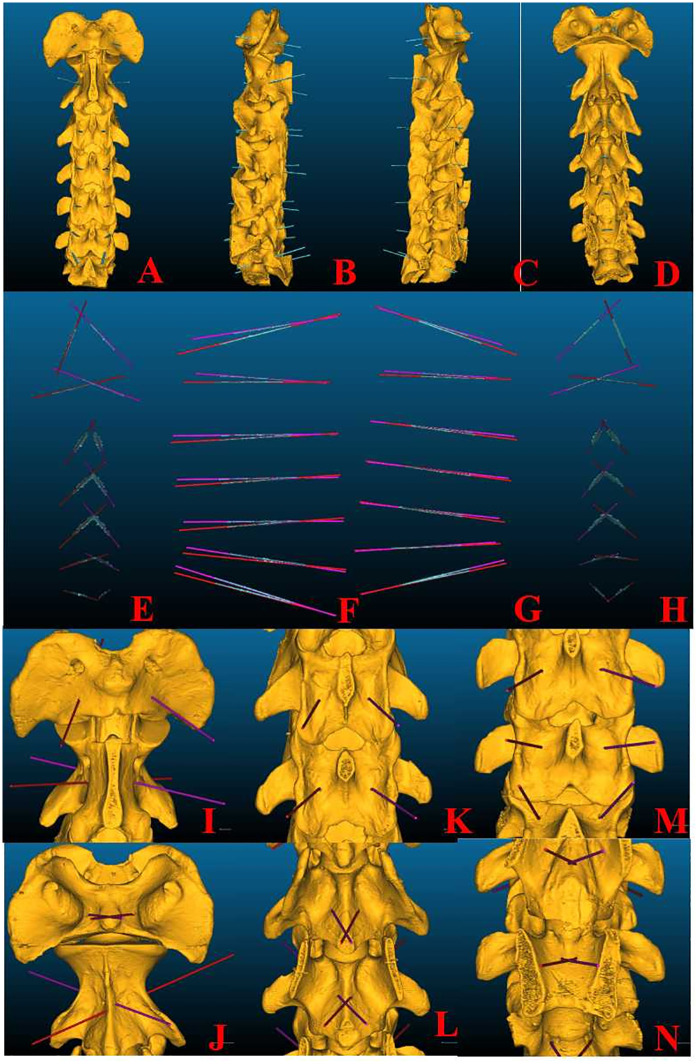
Distribution of the Kirschner Wire, Preoperative Designed Channels, and Postoperative Needle-Removed Skeleton. **A-D** Four views of the Kirschner wire on the postoperative needle-removed skeleton; **E-H** Four views of the preoperative designed channels and the distribution of the Kirschner wire; **I-N** Posterior-anterior views of the preoperative designed channels on the postoperative needle-removed skeleton: **I** Dorsal side of C1-2;J Ventral side of C1-2;K Dorsal side of C3-4; **L** Ventral side of C3-4; **M** Dorsal side of C5-7; **N** Ventral side of C5-7.

Fig 11 shows the distribution of the positions of the designed channels and Kirschner’s needles on the bone after the operation.

### Intraoperative RMS and registration accuracy data

The average RMS and registration accuracies were 0.424 ± 0.12 mm and 0.01 ± 0.008 mm, respectively, demonstrating that the registration error in this study was minimal, with high precision achieved at approximately 0.01 mm (Table 1).

**Table 1:** RMS and registration accuracy data.

Subject	RMS	Registration Accuracy (mm)
Model 1	0.41	0.005
Model 2	0.346	0.024
Model 3	0.422	0.025
Model 4	0.387	0.011
Model 5	0.44	0.001
Model 6	0.73	0.001
Model 7	0.4	0.007
Model 8	0.361	0.011
Model 9	0.328	0.007
mean ± SD	0.424 ± 0.12	0.010 ± 0.008

### In and out of the needle point offset value and in and out of the needle offset angle

The deviations of the entry point and the exit point were 0.904 ± 0.419 mm and 1.253 ± 0.607 mm, respectively. The angular deviation between the designed channel and the Kirschner needle was 0.918 ± 0.421 degrees, both before and after the operation. These results indicate that the pedicle punctures in this study exhibited minimal deviation and high precision (Table 2).

**Table 2:** needle point offset and angle offset.

Subject	Neo grade	injection point offset	exit point offset	angle offset	failure
Model 1	0	1.197	1.265	0.856	0
Model 2	0	1.192	1.244	1.139	0
Model 3	0	0.725	1.240	0.844	0
Model 4	0	0.923	1.127	0.920	0
Model 5	0	0.736	1.373	0.809	0
Model 6	0	0.742	1.266	0.916	0
Model 7	0	1.012	1.208	1.018	0
Model 8	0	0.832	1.247	0.961	0
Model 9	0	0.822	1.309	0.833	0

### The difference in the axis coordinate values (x, y, z) at the needle entry and exit points before and after operation

There was no significant difference in the average X-axis coordinate between the preoperative and postoperative entry points, with a mean difference of 0.044 ± 0.504 (*P* = 0.329). There was a significant difference in the average Y-axis coordinate difference, with a mean difference of 0.111 ± 0.446 (*P* = 0.006). There was also a significant difference in the average Z-axis coordinate, with a mean difference of 0.225 ± 0.694 (*P* < 0.001).

There were no significant differences in the X-axis coordinate difference (mean = 0.083 ± 0.736, *P* = 0.212), whereas significant differences were found in the Y-axis (mean = 0.248 ± 0.772, *P* < 0.001) and Z-axis (mean = −0.204 ± 0.839, *P* = 0.008) coordinate differences between the preoperative and postoperative needle exit points.

## Discussion

### Spinal pedicle screw placement navigation technology and structured light navigation

Minimally invasive, digital, and intelligent techniques are the hallmarks of modern spinal surgery. Intelligent planning for pedicle screws is key for the future of fully automated robotic surgery. The evolution of pedicle screw placement in spinal surgery has progressed from manual techniques to computer-assisted navigation systems and robotic navigation-assisted screw placement, continuously advancing toward digitalization, intelligence, minimal invasiveness, and precision.

At present, research has focused on the accuracy of nail placement, radiation damage, learning curve, etc. [28–31]. Computer navigation aids in selecting the appropriate screw size, improving fixation strength, reducing the intraoperative radiation dose and surgical time, and enhancing the surgical skills of spine surgeons in the early stages of the learning curve or those who infrequently perform such procedures [32,33]. Navigation assistance can greatly improve surgical outcomes, particularly in complex procedures such as cervical pedicle screw fixation, by increasing precision, accuracy, and consistency [34].

Computer navigation technology encompasses various methods, including near-infrared light navigation, electromagnetic navigation, laser navigation, and structured light navigation, which are distinguished by their core components used for surgical tracking and positioning. Electromagnetic navigation is particularly vulnerable to interference from magnetic fields present in the surgical environment, while laser scanning poses challenges due to radiation exposure and results in sparse point clouds, leading to relatively prolonged registration times. Near-infrared light navigation has emerged as the predominant choice in clinical applications owing to its established reliability [16]，however, it necessitates the use of additional reference frames or surface markers, the registration process requires fiducial markers，which complicates the overall process.

The accuracy of the final positioning in a navigation system is closely related to the precision of the registration process. Navigation based on structured light employs a markerless registration technique, utilizing hundreds of thousands to millions of point clouds, which enhances accuracy. This approach significantly reduces the time and frequency of intraoperative fluoroscopy, and in some cases, eliminates the need for it altogether, as it does not require reference frames. Furthermore, it enhances the workflow and efficiency of intraoperative navigation [35–38]. However, previous research on structured light navigation has predominantly concentrated on static structured light and has primarily been applied in neurosurgery, exemplified by the SINO neurosurgical robot [24].

This study employs a single dynamic structured-light navigation system for minimally invasive spinal puncture, offering distinctive features and advantages. Compared with near-infrared navigation, the differences and benefits are: (1) higher navigation accuracy; (2) markerless registration; (3) no need for additional dynamic reference frames; (4) reduced radiation exposure; (5) lower navigation costs. Compared with other structured-light navigations used in spinal surgery research, the differences and advantages are: (1) only a single navigation light source is used; (2) dynamic structured-light technology is employed; (3) it is suitable for minimally invasive surgery.

### Outcomes of qualitative and quantitative evaluation

The results of this study using dynamic structured light navigation for cervical pedicle puncture are as follows: the Neo classification and evaluation of all postoperative Kirschner wires at the pedicle position were rated as level 0. The intraoperative registration accuracy and RMS values were 0.01 mm and 0.42 mm, respectively. The deviation of the needle tip at the entry point before and after the operation was 0.904 mm, whereas the overall deviation at the exit point was 1.253 mm, and the deviation angle of the needle insertion and withdrawal was 0.918 degrees. The results indicated that the puncture effect was satisfactory in the qualitative evaluation. In the quantitative analysis, the registration accuracy was high, and the degree of puncture error was minimal. The navigation methods of scene scanning, static registration, and dynamic tracking of nailing instruments in single structured light surgery are feasible, with a low learning curve. This achieves the research goal of accurate and minimally invasive puncture navigation. It provides excellent theoretical and practical references for further research or studies by others.

### Comparison with other technologies

Qualitative evaluation has long been considered the clinical “gold standard” for assessing the accuracy of pedicle screw placement [39]. Most evaluations of screw placement effectiveness are based on qualitative assessment. For example, a total of 317 pedicle screws from C2 to C7 were implanted via O-arm-based 3D navigation, with screw accuracy rates of 100% and 96.2%, respectively, according to the Neo classification. Grade 1 screws accounted for 3.8% of the screws. However, metal-induced scattering artifacts from localized screws can affect the results of qualitative evaluation.Volk *et al*. [40] emphasized the necessity of quantitatively evaluating the effectiveness of accurately placing pedicle screws via navigation.

A meta-analysis involving six studies (including two controlled trials) demonstrated that a total of 482 cervical screws were placed using surgical robots (TiRobot, Cirq, Mazor X Stealth), of which 78.6% were CPSs. Among the 482 cervical screws, 471 (97.7%) achieved clinically acceptable levels (with cortical breach less than 2 mm), resulting in an average screw deviation of 0.95 mm. The acceptance rate for CPS was 96.9% [41]. The CUVIS spinal robot was used to place 40 screws in three human cervical spine specimens. The total insertion deviation was 1.08 ± 0.83 mm, the exit deviation was 1.86 ± 0.50 mm, and the angular deviation was 2.14 ± 0.77° [42]. Augmented reality (AR)-guided screw placement was performed on five human cadaveric cervical spine specimens, with 41 screws inserted from C3 to C7. The apex and trajectory errors in the axial plane were 1.25 mm and 1.8°, respectively, whereas the apex and trajectory errors in the sagittal plane were 1.39 mm and 1.77°, respectively. The GR classification for screws in grades A and B was 100% [43].

Zhu [44] employed a single static structured light scan to simultaneously acquire point clouds of both the screw placement instrument and the dorsal surface of the vertebral body. By separately registering the two point clouds, the surgical trajectory, along with the position and orientation of the screw placement instrument, was determined, generating motion commands for the robotic arm. Subsequently, drilling experiments for pedicle screw placement and accuracy validation were performed on a cervical spine model. In the drilling experiment, all 42 screws successfully passed through the pedicle. The average positional error at the entry point was 0.28 ± 0.16 mm, and the average angular error was 0.49 ± 0.24°. The criteria for successful cervical screw placement are an angular error of less than 3° and a tip position error of less than 3 mm [3]. Although this research method did not involve the placement of pedicle screws, the findings demonstrated a high level of puncture precision. It is anticipated that, in future experiments involving the insertion of pedicle screws, the accuracy of screw placement will align with the results documented in the literature for other image-guided and robot-assisted computer-assisted spinal CPS placements, all of which demonstrate significant deviations of less than 3°and 3 mm. These values fall well within the acceptable absolute navigation accuracy limits established by the FDA [43,45].

### Factors contributing to high-precision puncture

The calculation of bias varies across different studies. This research integrates qualitative analysis and quantitative evaluation, with results all falling within the range reported in the literature and at an excellent level. Image-guided navigation and surgical planning represent the leading edge in defining digital surgery [46].This study applied the concept of digital surgery to achieve high-precision puncture outcomes.

Mainly Related to the Following Factors: (1) Advantages of High-Precision 3D Structured-Light Cameras: The camera was fixed on top of a post at a height of 2 m, with a spatial resolution of 3 megapixels. Its working distances were set to 0.9–2.9 m, and the field of view was set to 800 mm * 605.8 mm at 1.5 m. Unlike static structured light, which captures only single-frame images with low precision, this camera projects a series of dynamic structured light. It scans a wide range with deep depth, a high frame rate, and a high transmission rate, instantly acquiring a high-density point cloud containing millions of points on the model surface for registration, thereby significantly enhancing registration accuracy. 3D structured light facilitates registration with the external body surface by capturing point clouds (static registration). This technology also allows for large-scale, direct dynamic tracking of insertion instruments. Within the same world coordinate system as the structured light, navigation is implemented rapidly, which enhances the final accuracy of screw placement. By minimizing navigation steps, employing a straightforward coordinate transformation chain, reducing cumulative navigation errors, and avoiding the various drawbacks associated with placing reference frames, this method enables minimally invasive puncture procedures without the necessity for open surgery.

(2) The RCCS has the following characteristics. ①The developed cylindrical fitting navigation channel algorithm can accurately generate a cylinder and transform it into a navigation channel by scanning only a portion of the drill sleeve surface with structured light. Moreover, the algorithm performs cylindrical fitting only for drill sleeves with a diameter of approximately 25 mm within the structured light scanning range, ensuring high fitting accuracy. When light source occlusion occurs, the system can remove the obstruction and continue navigation without being affected by prolonged or small occlusions. This process eliminates the need to restart the structured light scan or perform additional image registration and instrument calibration. ②One-time registration enables large-scale, long-segment, multidirectional navigational punctures. It simplifies the operation, reduces the complexity of the navigation steps, and minimizes the accumulation of errors across multiple stages. ③Surgical planning, navigation implementation, and data analysis are all integrated within a single RCCS. ④The purely three-dimensional nature of surgical planning and navigation allows for detailed and clear observation of both the surface and internal structure of the 3D model. This approach is particularly advantageous for complex designs, such as bovine cervical vertebrae with numerous cavities. This approach significantly reduces the risk of inadvertent perforation, prevents intraoperative mispunctures, and enhances puncture accuracy. The three-dimensional nature of the software interface closely resembles reality, offering a viewing experience that aligns more closely with human visual perception. During the procedure, it is feasible to temporarily modify the channel design and adjust the puncture trajectory as necessary. Intraoperative navigation software can be designed to accommodate these changes. This study employs dynamic structured light, which dynamically fits a cylindrical model to generate a new navigation puncture direction, thereby meeting the requirements for the revised puncture trajectory. During the procedure, it is entirely feasible to temporarily alter the channel design, adjust, or correct the puncture trajectory as necessary. The intraoperative navigation software can be designed on the fly, as this study utilizes dynamic structured light, which dynamically fits a cylinder to generate new navigation puncture directions, thereby meeting the new puncture requirements. ⑤The left and right window interfaces of the RCCS offer distinct viewing perspectives within the same coordinate system. Each window serves a specific function: the left window is dedicated to trajectory design and navigation puncture, whereas the right window is used for tracking nail placement instruments and real-time monitoring of the cylinder fitting effect. The design of the left and right windows can reduce mutual interference, prevent major errors and omissions, and improve the navigation puncture effect.

(3) The adjustment device has the following characteristics. The adjustment device precisely positions the navigation drill sleeve through simple actions of rotating the turntable, adjusting the telescopic rod, and sliding the magnetic base. It generates the navigation channel quickly through structured light scanning without the need for coordinate system transformation. During the procedure, the navigation channel can be easily and rapidly adjusted to align with the preoperative design trajectory.

(4) The Puncture instruments have the following characteristics. Puncture instruments feature high-precision machining, ensuring that the navigation drill sleeve, puncture tube, and Kirschner wire maintain excellent coaxiality. The center of the navigation drill sleeve serves as the working channel, enabling rapid and precise puncture without the need for additional nail instrument tracers. The inner sleeve of the puncture tube reaches the bone surface, whereas the outer sleeve enters the skin, ensuring the safe passage of the Kirschner wire through the pedicle.

Limitations and Prospects. This study has several limitations: (1) No human specimen trials were conducted. The bovine cervical vertebrae are larger, whereas human vertebrae are relatively smaller, which may lead to discrepancies in puncture accuracy. The bovine cervical vertebrae model fails to replicate the typical physiological cervical lordosis. Given the angulation and anatomical characteristics of human vertebrae, this may diminish the accuracy of screw placement in clinical patients. (2) No in vivo animal experiments were conducted, and the clinical surgical environment was not fully simulated. This study was performed under controlled laboratory conditions, without respiratory motion, bleeding, or anatomical variation, and cannot reproduce the complexities of an actual surgical procedure, such as intraoperative movements, bleeding, tissue contraction, or intraoperative tissue deformation. (3) The absence of a control group prevents any comparative analysis of the advantages and disadvantages of this navigation system in relation to others. (4) Only pedicle puncture was performed; a guiding needle was placed, and pedicle screws were not inserted. (5)Software aspects:the current RCCS V1.0 is designed for principle verification and has not yet undergone modular assembly. There are limitations in terms of software interactivity; for example, the entry and exit needle points are marked with a single color, making it visually challenging to distinguish them during comparison. Currently, the RCCS navigation software is only in version 1.0. With the addition of modules, software updates, and improvements, versions such as 2.0 and 3.0 will gradually be released. Second, third-party software is currently required for 3D reconstruction. Development is underway for a 3D reconstruction module based on AI technology, which features automatic filling and segmentation capabilities. (6) Regulating instruments and puncture instruments:The current system relies on manual operation for instrument adjustments and puncture procedures, lacks mechatronic control and integration of electric devices, and does not incorporate a robotic arm or supporting software into the RCCS system.

The structured-light camera used in this experiment had a working range of 0.9 to 2.9 m, which fully meets the experimental requirements. With advancements in high-resolution technologies such as Digital Micromirror Devices (DMD), Liquid Crystal Displays (LCD), Liquid Crystal on Silicon (LCoS), and Micro-Electro-Mechanical Systems (MEMS), structured light will be able to effectively address the requirements for wide field-of-view and long-distance navigation. As with other optical navigation systems, light source occlusion must be considered. However, structured light can integrate active calibration protocols to enable rapid intraoperative re-registration [26], ensuring the continuity and accuracy required for surgical navigation. A key advantage of structured light is its ability to perform repeated and continuous scanning without any risk of radiation exposure.

Intraoperatively, when image drift occurs due to frame loosening (which can be detected by probes indicating whether certain anatomical landmarks are in the correct positions), the navigation system can re-scan with structured light and perform real-time re-registration using specialized algorithms. This response mechanism, enabled by the properties of structured light, is fast and effective, avoids radiation exposure, and can be repeated multiple times for re-registration. The integration of medical and engineering disciplines, coupled with the advancement of industry‒education collaboration, is poised to enhance the field of medicine through the adoption of new technologies. Image-guided navigation is leading the way for future innovations in spinal surgery, providing increased precision and facilitating progress [47]. Furthermore, advancements in technologies such as DLP chips and 3D structured light cameras will yield higher resolution, broader scanning capabilities, and faster processing speeds. These developments will enable the creation of precise, efficient, and cost-effective surgical tools and software specifically designed for minimally invasive spinal surgeries.

## Conclusion

In laboratory conditions,the single dynamic structured light navigation system can simultaneously perform preoperative complex design, intraoperative scene scanning, static registration, and dynamic tracking of the piercing instruments, achieving high-precision minimally invasive puncture. In the future, the surgical environment should be simulated to conduct further research and explore its effectiveness in surgical applications under complex conditions.

## Supporting information

S1 File Data collection for each model. (RAR)

## Abbreviations

RMS: root mean square
CPS: cervical pedicle screws
MIS: Minimally invasive spine surgery
AR: Augmented reality
LCD: Liquid Crystal Displays
LCoS: Liquid Crystal on Silicon
MEMS: Micro-Electro-Mechanical Systems
ICP: Iterated Closest Points
DMD: Digital Micromirror Devices
